# Medical Items

**Published:** 1908-07

**Authors:** 


### MEDICAL ITEMS

PROSTATIC IRRITATION.—The influence of residual urine in setting up prostatic inflammation is well known. When the urine is concentrated or unduly acid it becomes doubly irritating. To induce a bland, free, unirritating urine is to remove a common exciting cause of the trouble. For this purpose there is no better remedy than Alkalithia. Shut off the use of rhubarb, tomatoes and strawberries.

---

### THE BORDERLAND OF DISEASE

There is a growing tendency on the part of medical men to recognize the pathological importance of certain, at present, little understood conditions of the blood. Some of these indeterminable deviations from the normal present none of the aspects of the anemias, but nevertheless bear a direct relation to increased susceptibility to bacterial infection. The studies of Wright on the opsonins, so called, are of special interest in this direction, inasmuch as they have in a measure converted many of our abstract theories into concrete facts. That certain constituents of the blood may be diminished without apparent decrease of the corpuscular elements or of the hemoglobin, is at last fairly well established, and while the specific properties of these constituents are not as yet definitely known, there is abundant reason for attributing certain phases of malnutrition, as well as a general lowering of organic resistance to bacteria, to their absence or decrease. The clinical expression of this blood weakness, or chemico-physiologic deficiency, is subject to great variation, but the symptom-complex usually consists of a general physical decline, loss of weight, increased tendency to fatigue, and a fickle

